# Diagnostic Value of Gauze Filtration Technique: A Comparison with Conventional Methods in a Diagnostic Laboratory in Pakistan

**DOI:** 10.7759/cureus.3615

**Published:** 2018-11-19

**Authors:** Naima Maqsood, Aymen Shakeel, Najia K Ghanchi, Ahmed Raheem, Feroz Zaheruddin, Ghazala Jabeen, Afsheen Raza, Mohammad A Beg

**Affiliations:** 1 Internal Medicine, Aga Khan University Hospital, Karachi, PAK; 2 Family Medicine, Aga Khan University Hospital, Karachi, PAK; 3 Pathology, Aga Khan University Hospital, Karachi, PAK; 4 Pathology, Dadabhoy Institutes of Higher Education, Karachi, PAK

**Keywords:** intestinal parasites, gauze filtration technique, direct wet mount, sedimentation technique, diagnostics, pakistan

## Abstract

Background

Intestinal parasites cause significant morbidity and impact human development with an enormous global burden. Diagnosis of intestinal parasites by conventional methods has several limitations. The gauze filtration technique is a relatively simple method that has been shown to identify intestinal parasites with a high sensitivity and specificity. The aim of this study was to determine the diagnostic value of this technique as compared to more conventional methods in a large acclaimed laboratory within Pakistan.

Methods

A total of 50 stool samples collected for routine diagnostic workup from patients age between 2-70 years were collected from the parasitology section of the Aga Khan University Hospital Clinical Laboratory. A direct wet mount, sedimentation technique, and gauze filtration technique were performed on all of the stool samples, and the sensitivity, specificity, negative predictive value, and positive predictive value were analyzed.

Results

It was observed that the number of organisms observed by gauze filtration as compared to direct wet mount and sedimentation technique was higher for *B. hominis*, *G. lamblia* cysts and trophozoites, and *I. bütschlii*. Also, the detection rate was significantly higher for *B. hominis* and *G. lamblia* cysts using the gauze filtration technique. The sensitivity and specificity of the gauze filtration technique were found to be 95.8% and 100%, respectively.

Conclusion

There is a significantly better stool sample parasite detection rate using the gauze filtration technique as compared to the conventional sedimentation techniques. The utility of the gauze filtration technique seems economically and technically feasible for diagnostic laboratories in resource-limited settings.

## Introduction

Intestinal parasitic infections (IPI) are a common cause of morbidity worldwide. According to the World Health Organization (WHO), around 3.5 billion people harbor parasites, 450 million are ill as a result of these infections, and more than 880 million children are in need of treatment for these parasites [1]. The World Development Report ranked intestinal parasitism as a major cause of disease burden in children aged 5 – 14 years. Although a serious global burden, intestinal parasites are neglected and can be treated by cost-effective medication following diagnosis [2].

In Pakistan, the burden of intestinal parasitic infections is high as unhygienic sanitary conditions and the contaminated water supply serve as a constant source of infection for these parasites. Though highly burdened, the prevalence rate reported varies depending on the area of study, as well as the study participant. Most studies were conducted on a small scale, either in hospitals or schools from slum urban or rural areas [3-5]. Large-scale surveys have not been performed as yet. However, laboratories in Pakistan report the burden of intestinal parasites to be high (with an estimated prevalence to be as high as 47 - 52.8% in Karachi) with Giardia lamblia reported as the most prevalent organism, followed by *Blastocystis hominis*, *Ascaris lumbricoide*s, and *Hymenolepis nana* [6-7]. Studies conducted in other areas of Pakistan reported similar trends of IPI prevalence, ranging from 20 - 51.5% [3-6, 8].

Identification and diagnosis of intestinal parasites are challenging for laboratories as conventional methods demonstrate various limitations. Briefly, conventional methods for diagnosis of intestinal parasitic infections from stool samples are based on direct wet mount and concentration techniques, such as flotation and sedimentation. In a direct wet mount, protozoan trophozoites, cysts, oocysts, helminth eggs, and larvae may be seen and identified by microscopic examination. A small amount of stool specimen is placed on a microscope slide, mixed with saline, and a coverslip is placed. The entire coverslip area is scanned using the 10X objective for the detection of trophozoites, cysts of protozoa, and eggs and larvae of helminths. In concentration techniques, flotation techniques use solutions which have higher specific gravity than the organisms that float so that the organisms rise to the top and the debris sinks to the bottom. The main advantage of this technique is that it produces cleaner material than the sedimentation technique. The disadvantages of most flotation techniques are that the walls of the eggs and cysts will often collapse, thus hindering identification. Also, some parasite eggs do not float. On the other hand, sedimentation techniques use solutions of lower specific gravity than the parasitic organisms, thus concentrating the latter in the sediment [9].

The major technical limitations associated with concentration techniques include low parasite density in feces, variations in fecal consistency, the collapse of the walls of eggs and cysts during flotation, and the inability of some parasite eggs to float [10-11]. Keeping these limitations in mind, it is imperative to study and test new methodologies that may address these limitations and increase the diagnostic yield of IPI testing. The aim of this study was also to demonstrate the economic feasibility of such techniques so that routine diagnostics can be improved.

In 1978, the gauze filtration technique, also known as the spontaneous sedimentation technique in a tube (SSTT), was described to be sensitive and cost-effective for diagnosis of intestinal parasites [12]. Briefly, the technique utilized filtration of homogenized patient stools with surgical gauze, followed by sedimentation. The technique is well documented in studies to show a high diagnostic value, as compared to the wet mount and sedimentation techniques [10, 13-15]. However, this technique has not, as yet, been tested for use for diagnostic purposes in Pakistan. Therefore, the aim of this study was to compare and document the diagnostic value of three different techniques, i.e, the conventional direct wet mount, sedimentation, and the gauze filtration technique for their sensitivity and specificity, and to detect intestinal parasitic infections in a high throughput parasitology diagnostic laboratory in Karachi, Pakistan.

## Materials and methods

Study design and settings

This cross-sectional study was conducted at the Department of Pathology and Laboratory Medicine of The Aga Khan University Hospital, Karachi from March 2015 through September 2015. A total of 50 routine diagnostic samples age between 2-70 years were included in the study. All three techniques, i.e., direct wet mount, sedimentation techniques (Mini-system Paragreen) (Biolife Italiana Srl, Milan, Italy), and gauze filtration were performed on human stool samples.

Diagnostic techniques for diagnosing parasites

For a direct wet mount, one drop each of saline and iodine (Lugol’s solution) was placed on both edges of the slide. The fecal specimen (50 µL) was emulsified in the saline and iodine with an applicator stick and covered with coverslips on either side of the slide. The slide was examined under 10X and 40X magnification.

Sedimentation was performed initially using the Mini-system Paragreen. For each fecal specimen, approximately 5 grams was taken with a spatula, transferred into the collection tube, and shaken vigorously for 30 seconds. The bottom cap was then replaced with the sedimentation tube, keeping the vial upside down. The entire system was centrifuged for three minutes at 2,000 rpm. The supernatant was discarded, along with the collection tube, and the sediment was re-suspended with one to two drops of saline and examined under the microscope, first at 10X and then at 40X magnification. One drop of Lugol’s iodine was added to the slide to enhance the visibility of parasites.

**Figure 1:**
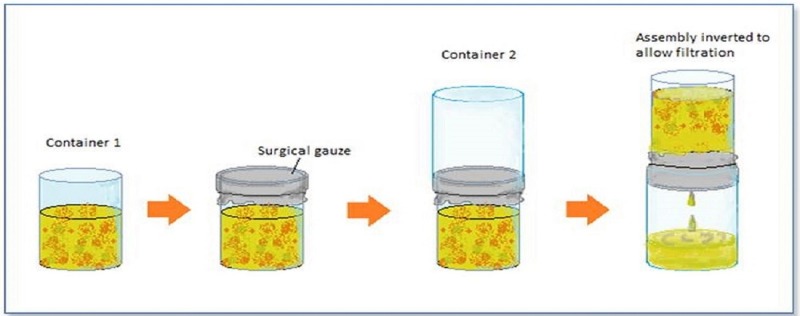
Gauze filtration technique

To perform gauze filtration, the consistency of the stool samples has to be taken into consideration. Formed stools were mashed, while normal saline was added to loose stools. For every gram of feces, 3 ml of saline was added to attain the desired consistency. A spatulaful (approximately 5 grams) of stool was then taken from each sample and placed in Container 1 (Figure 1). Standard 10 cm × 10 cm (8-ply) surgical gauze was used. Pore size not stated by the manufacturer. The sterile surgical gauze (4" X 4"), folded in half to make a double layer, was placed over the container and a thread was tied around it to hold it in place. Container 2, having a wider mouth than Container 1, was inverted and fitted over the mouth of Container 1. The entire assembly was then inverted and given adequate time (10 - 15 minutes on average) to allow filtration of the suspension through the surgical gauze. Once the filtration had taken place, the solid debris remained in Container 1 as residue and the filtrate was collected in Container 2. A wet mount was prepared from the filtrate, transferred onto a slide using a dropper, stained with iodine, and observed under a light microscope under 10X and 40X magnification. If no parasites were observed, the filtrate was centrifuged at 1,000 rpm for three minutes and the supernatant was discarded, while the residue was again placed on a slide screened for parasites using a stool wet mount. Overall, comparing both sedimentation technique to the wet preparation is important as this is the technique is widely used in laboratories in the developing world.

Characterization of organisms

Organisms observed were characterized as scanty/occasional, few, or moderate, depending on the number of organisms seen per field. An observation was labeled as “rare” if only one organism was seen per field, “few” if two to nine organisms per field were observed, and “moderate” if 10 - 25 organisms per field were observed.

Statistical analysis

Data were analyzed using the IBM Statistical Package for Social Sciences (SPSS) Statistics for Windows, Version 21.0. (IBM Corp., Armonk, NY, US). Mean ± standard deviation (SD) or median (interquartile range = IQR) was evaluated for quantitative variables as appropriate. Frequency and percentage were computed for each different organism and also for the type of organism detected by each technique. Fisher's exact test or Chi-square test was used to check the association between the various organisms detected by each technique. A 2 x 2 table was constructed and sensitivity, specificity, positive predictive value, negative predictive value, and overall accuracy were determined for sedimentation and gauze filtration techniques. The sedimentation technique was used as a reference standard. P-value < 0.05 was considered significant at 95% confidence interval (CI).

## Results

The results of the study compared the number of organisms observed by each technique, i.e., gauze filtration technique, direct wet mount, and sedimentation technique, and determined the sensitivity of the sedimentation and gauze filtration technique. It was observed that a significant difference in the detection rates exists for cysts of *B. hominis* and *G. lamblia* using the gauze filtration technique (Table 1).

**Table 1:** Detection Rate of Direct Wet Mount, Sedimentation and Gauze Filtration Technique

Organisms	Techniques	Detection rate								P value
		None		Rare		Few		Moderate		0.295
		Count	%	Count	%	Count	%	Count	%	
Entamoeba coli	Wet	47	94.0%	0	0.0%	3	6.0%	0	0.0%	
	Sedimentation	50	100.0%	0	0.0%	0	0.0%	0	0.0%	
	Gauze	47	94.0%	0	0.0%	2	4.0%	1	2.0%	
B. hominis	Wet	31	77.5%	3	7.5%	6	15.0%	0	0.0%	0.049*
	Sedimentation	40	80.0%	3	6.0%	7	14.0%	0	0.0%	
	Gauze	33	66.0%	1	2.0%	11	22.0%	5	10.0%	
G. lamblia cyst	Wet	37	74.0%	0	0.0%	13	26.0%	0	0.0%	0.000*
	Sedimentation	42	84.0%	4	8.0%	4	8.0%	0	0.0%	
	Gauze	37	74.0%	0	0.0%	6	12.0%	7	14.0%	
I. butschlii	Wet	44	88.0%	0	0.0%	6	12.0%	0	0.0%	0.114
	Sedimentation	47	94.0%	1	2.0%	1	2.0%	1	2.0%	
	Gauze	45	90.0%	0	0.0%	2	4.0%	3	6.0%	
Ancylostoma duodenale	Wet	49	98.0%	0	0.0%	1	2.0%	0	0.0%	1.000
	Sedimentation	49	98.0%	0	0.0%	1	2.0%	0	0.0%	
	Gauze	49	98.0%	0	0.0%	1	2.0%	0	0.0%	
Cystoisospora	Wet	48	96.0%	2	4.0%	0	0.0%	0	0.0%	1 .000
	Sedimentation	48	96.0%	2	4.0%	0	0.0%	0	0.0%	
	Gauze	48	96.0%	2	4.0%	0	0.0%	0	0.0%	
G. lamblia trophozoite	Wet	49	98.0%	0	0.0%	1	2.0%	0	0.0%	0.602
	Sedimentation	50	100.0%	0	0.0%	0	0.0%	0	0.0%	
	Gauze	49	98.0%	0	0.0%	1	2.0%	0	0.0%	

The sensitivity, specificity, negative predictive value (NPV), and positive predictive value (PPV) of the gauze filtration and the sedimentation techniques are shown in Tables 2-3 accordingly. The overall sensitivity for the gauze filtration and sedimentation technique was found to be 95.8% and 58.3%, respectively, while the specificity was found to be 100% for both techniques.

**Table 2:** Sensitivity and Specificity of Gauze Filtration Technique P-values are based on Chi-square test NPV: negative predictive value; PPV: positive predictive value

Organism	Sensitivity	Specificity	PPV (%)	NPV (%)	Accuracy (%)	P-value
E. coli	1.00 (0.44 - 1)	1.00 (0.92 - 1.00)	100	100	100	P < 0.001
B. hominis	0.89 (0.69 - 0.98)	1.00 (0.89 - 1.00)	100	93.9	96	P < 0.001
G. lamblia cyst	1.00 (0.77 - 1.00)	1.00 (0.91 - 1.00)	100	100	100	P < 0.001
I. butschlii	0.83 (0.44 - 0.97)	1.00 (0.92 - 1.00)	100	97.7	98	P < 0.001
Ancylostoma duodenale	1.00 (0.20 - 1.00)	1.00 (0.92 - 1)	100	100	100	0.02
Cystoisospora	1.00 (0.21 - 1.00)	(0.93 - 1.00)	50	100	98	0.02
Cryptosporidium	1.00 (0.21 - 1.000)	1.00 (0.93 - 1.00)	100	100	100	0.02
G. lamblia trophozoite	1.00 (0.21 - 1.00)	1.00 (0.93 - 1.00)	100	100	100	0.02
Overall	0.96 (0.86 - 0.99)	1.00 (0.99 - 1.00)	100	99.5	99.5	P < 0.001

**Table 3:** Sensitivity and Specificity of Sedimentation Technique Mini-system Paragreen P-values are based on the Chi-square test NPV: negative predictive value; PPV: positive predictive value

Organisms	Sensitivity	Specificity	PPV (%)	NPV (%)	Accuracy (%)	P value
*E. coli *	1.00 (0.44 - 1.00)	1.00 (0.92 - 1)	100	100	100	< 0.001
*B. hominis*	0.34 (0.20 - 0.53)	1.00 (0.85 - 1)	100	52.5	62	0.003
*G. lamblia cyst*	0.62 (0.36 - 0.83)	1.00 (0.91-1)	100	88	90	< 0.001
*I. butschlii*	0.5 (0.19 - 0.81)	1.00 (0.92 - 1)	100	93.6	94	< 0.001
*Ancylostoma duodenale*	1.0 (0.21 - 1.00)	1.00 (0.93 - 1)	100	100	100	0.020
*Cystoisospora*	1.00 (0.34 - 1.00)	1.00 (0.93 - 1)	100	100	100	<0.001
*Cryptosporidium*	1.00 (0.21 - 1.00)	1.00 (0.93 - 1)	100	100	100	0.020
*G. lamblia trophozoite*	----	----				
Overall	0.58 (0.43 - 0.71)	1.00 (0.99 - 1)	100	95.2	95.5	< 0.001

## Discussion

Our study aimed to compare the gauze filtration technique with the gold standard sedimentation technique used routinely in our parasitology diagnostic laboratory in Karachi, Pakistan. The results showed an increased sensitivity of the gauze filtration method compared to routine diagnostic methodologies used to identify intestinal parasites.

Direct wet mount visualization of the stool samples for microscopic analysis is the key step in detecting parasites infecting the gastrointestinal tract, as well as for quantifying fecal leukocytes and red blood counts (RBCs). For a conventional wet mount, two preparations are made for stool samples; one with normal saline and another with iodine stain. The direct wet mount is a rapid and straightforward technique requiring relatively inexpensive materials and minimum expertise. It allows a rough estimation of the parasitic load and determination of the developmental stage of the parasitic species. Characteristic motility of trophozoites can also be appreciated [9, 11]. However, there are some limitations to the direct wet mount technique. Normal saline and iodine used for preparation dry up within a few minutes, rendering the slide unreadable. Furthermore, specimens cannot be preserved by this method so a fresh slide has to be prepared every time the sample needs to be analyzed, which takes up considerable resources as well as time. Since this technique does not involve a concentration method, it is likely that a sample with a low parasitic load will be reported negative, thus reducing the sensitivity of this method [11, 13].

Over the last few decades, several new methods have been employed with the aim of improving the diagnostic yield of the traditional wet mount technique. The sedimentation technique is a widely used diagnostic tool in the detection of parasitic protozoa and helminths from infected stool samples. The most commonly used sedimentation techniques include the Mini-system Paragreen technique. In an endemic setting, variable sensitivities and specificities of sedimentation were observed with a high specificity of 98.8% but an unacceptably low sensitivity of 19.7% [16-17]. The variation in sensitivity and specificity of the sedimentation technique can be attributed to factors, such as the study setting (endemic vs non-endemic) and the type of sedimentation technique and organisms being detected. These low sensitivities could also be accounted for by the fact that the ova and parasitic species may not concentrate properly or may be damaged during centrifugation [10, 16, 18-20].

The sedimentation technique used in this study employed closed concentration systems that have been documented to have a brief processing time, a better diagnostic yield than direct microscopy, and a lower expertise requirement. Therefore, these systems can increase the diagnostic efficiency of laboratories processing a high number of samples. Furthermore, the Mini-system Paragreen uses a non-hazardous fixative which aids in the morphological preservation of the ova and parasites. However, its main limitation is that the fixative causes a hindrance in the identification of parasites (thus lowering the sensitivity), particularly for *Blastocystis hominis* and *Giardia lamblia* cysts [10, 16, 18-20].

There is a constant need for developing new methods that are more cost-effective and require less expertise whilst giving at least comparable levels of diagnostic accuracy. Therefore, our study compared a gauze filtration sedimentation technique with the routinely used sedimentation and wet mount techniques. The gauze filtration mainly functions to concentrate stool samples by removing fecal fat and saline from the sample, thereby increasing the chance of detection of ova and parasites from the concentrated stool [10, 12-13]. This increases the probability of the detection of ova and parasites in samples which may have a low parasitic load and are negative by a wet mount.

A recent study conducted by Tilak et al. compared the detection rate of the gauze filtration technique with the sedimentation and conventional wet mount techniques [13]. The results showed that the parasite detection rate of the gauze technique was 96.5%, as compared to the sedimentation technique, indicating the diagnostic efficacy of this method. Furthermore, it was also reported that the performance of the gauze sedimentation technique was comparable to the enzyme-linked immunosorbent assay (ELISA) and the real-time polymerase chain reaction (PCR)-based methods. A comparative study with other techniques, including Kato-Katz, direct smear, commercial kit, and sedimentation techniques, showed that the gauze method had a sensitivity of 95% [16-17].

Results from our study showed that compared to the sedimentation technique, the gauze filtration technique was able to detect *B. hominis *and *G. lamblia* efficiently with a sensitivity of 95.8% and a specificity of 100%. However, for other tested organisms, no significant difference was observed between the two techniques. Since these two organisms are highly prevalent in Pakistan, the gauze technique can potentially be useful in intestinal parasite diagnostics. The main advantages of utilizing this technique in routine diagnostics are that it is less time-consuming, requires only normal saline, organism morphology can be observed with ease as no formalin is used, gauze filtration removes debris making it a clear sample for handling and viewing, and it does not requires any specialized training [18]. Thus, it is a technique that can be helpful in diagnostic laboratories operating in high parasite burden regions of Pakistan, such as Karachi.

## Conclusions

There is a significantly better stool sample parasite detection rate using the gauze filtration technique as compared to the conventional sedimentation techniques. The utility of the gauze filtration technique seems economically and technically feasible for diagnostic laboratories in resource-limited settings.
